# Targeting EGR1 Attenuates *CCL2* Expression in Inflammatory Keratinocytes and Allergic Skin Inflammation

**DOI:** 10.3390/ijms27208973

**Published:** 2026-10-10

**Authors:** Yena Choi, Euitaek Jung, Junekyu Han, Soon Young Shin

**Affiliations:** 1 Department of Biological Sciences, College of Life Science, Konkuk University, Seoul 05029, Republic of Korea; yyenaa421@konkuk.ac.kr (Y.C.); mylife4sci@konkuk.ac.kr (E.J.); wnsrb0422@konkuk.ac.kr (J.H.); 2 Cancer and Metabolism Institute, Konkuk University, Seoul 05029, Republic of Korea

**Keywords:** Early growth response 1 (EGR1), CCL2, chemokine network, keratinocyte, skin inflammation, house dust mite, RNA sequencing, transcription factor inhibitor

## Abstract

Early growth response 1 (EGR1) is a zinc-finger transcription factor involved in inflammatory gene regulation, but the extent to which pharmacological inhibition of EGR1 DNA-binding activity influences inflammatory transcriptional responses in keratinocytes remains incompletely understood. Here, we explored the transcriptional effects of pharmacological EGR1 inhibition in tumor necrosis factor-α (TNFα)-stimulated HaCaT keratinocytes. RNA sequencing was employed as an exploratory, hypothesis-generating screen using three structurally related EGR1 inhibitors (IT21, IT23, and IT25) to identify candidate genes showing concordant expression patterns across the three pharmacological perturbations. This exploratory analysis suggested broader changes in inflammation-associated transcriptional programs and identified several leukocyte-recruiting chemokines among the candidate genes. *CCL2* was selected as a representative candidate for independent biological and mechanistic validation. IT25 treatment and EGR1 knockdown attenuated TNFα-induced *CCL2* expression, while promoter–reporter analysis, EGR1-binding site mutagenesis, EGR1 overexpression, and chromatin immunoprecipitation supported EGR1-dependent transcriptional regulation of *CCL2* in keratinocytes. Furthermore, topical IT25 treatment attenuated house dust mite-induced atopic dermatitis-like skin inflammation and was associated with reduced epidermal *CCL2* expression and inflammatory cell infiltration in vivo. Collectively, these findings establish EGR1-dependent regulation of *CCL2* in inflammatory keratinocytes and support further investigation of EGR1 DNA-binding activity as a pharmacological target in cutaneous inflammation.

## 1. Introduction

Chronic cutaneous inflammatory disorders, such as atopic dermatitis (AD) and psoriasis, are complex, multifactorial diseases characterized by persistent epidermal barrier disruption and sustained immune activation [1,2]. A major challenge in the treatment of these conditions is the functional redundancy and cross-talk among inflammatory mediators [3]. During disease progression, environmental allergens, pathogens, and mechanical stress can induce upstream inflammatory cytokines, including tumor necrosis factor-α (TNFα), which activate epidermal keratinocytes to produce a broad spectrum of secondary cytokines, epithelial alarmins, and leukocyte-recruiting chemokines [4,5].

Because multiple chemokines can engage overlapping receptors and signaling systems to promote leukocyte recruitment, therapeutic strategies targeting individual downstream mediators may provide incomplete control of complex inflammatory responses [5,6]. Targeting upstream regulatory mechanisms that influence multiple inflammatory mediators may therefore provide an alternative strategy for modulating these interconnected inflammatory pathways [6].

Early growth response 1 (EGR1) is a zinc-finger immediate-early transcription factor that is rapidly induced by diverse stimuli, including growth factors, mechanical stress, and pro-inflammatory cytokines such as TNFα [7,8,9]. EGR1 has been implicated in multiple inflammatory and pathological processes, including vascular inflammation, tissue remodeling, sensory neuroimmune communication, and macrophage activation [10,11]. Genetic deletion of *Egr1* in mice and shRNA-mediated silencing of EGR1 in human keratinocytes have been shown to reduce the expression of inflammatory mediators and attenuate experimental skin inflammation [11,12]. Previous studies have further established direct EGR1-dependent transcriptional regulation of individual inflammatory mediators, including *IL6*, *IL8*, and *CCL2*, in several non-cutaneous disease contexts [13]. Our previous studies have also demonstrated EGR1-dependent regulation of individual inflammatory genes in keratinocytes and characterized small-molecule compounds that interfere with EGR1 zinc-finger DNA-binding activity [11,12]. In particular, we previously developed and characterized a series of EGR1-targeting compounds, including IT21, IT23, and IT25, and demonstrated their anti-inflammatory effects in keratinocytes and an AD-like mouse model [12]. However, the downstream inflammatory transcriptional responses associated with pharmacological EGR1 targeting remained incompletely defined.

In the present study, we therefore employed RNA sequencing (RNA-seq) as an exploratory, hypothesis-generating transcriptomic screen in TNFα-stimulated HaCaT keratinocytes treated with IT21, IT23, or IT25. Given the exploratory nature of this analysis and the absence of independent biological replication for each sequencing condition, expression patterns observed across the three pharmacological perturbations were used for candidate prioritization rather than as evidence of reproducible differential expression. This analysis identified several leukocyte-recruiting chemokines among the candidate genes, and *CCL2* was selected as a representative candidate for independent biological and mechanistic validation. We examined whether the previously reported EGR1-dependent regulation of *CCL2* in breast cancer cells [13] also operates in inflammatory keratinocytes using pharmacological and genetic approaches, promoter–reporter analysis, EGR1-binding-site mutagenesis, and chromatin immunoprecipitation (ChIP). Finally, we examined *CCL2* expression and inflammatory cell infiltration following topical IT25 treatment in an independently performed HDM-induced AD-like mouse experiment. All experiments and datasets analyzed in the present study were independently generated and are distinct from those reported in our previous study [12].

## 2. Results

### 2.1. Exploratory Transcriptomic Profiling Identifies Candidate Inflammation-Associated Expression Patterns in Tnfα-Stimulated Keratinocytes

The small-molecule compounds IT21, IT23, and IT25 have previously been shown to interfere with the transcriptional activity of EGR1 by targeting its zinc-finger DNA-binding domain [12]. To explore the broader transcriptional responses associated with pharmacological EGR1 inhibition during keratinocyte inflammation, we performed QuantSeq 3′ mRNA sequencing in human HaCaT keratinocytes treated with vehicle control (Normal), TNFα (5 ng/mL) alone, or TNFα in combination with IT21, IT23, or IT25 (5 μM). The transcriptomic analysis was conducted as an exploratory, hypothesis-generating screen with one sample per experimental condition ( n = 1) and without independent biological replication. Accordingly, the sequencing data were interpreted descriptively and used for candidate prioritization rather than for statistical inference of differential expression or biological reproducibility.

**Figure 1:**
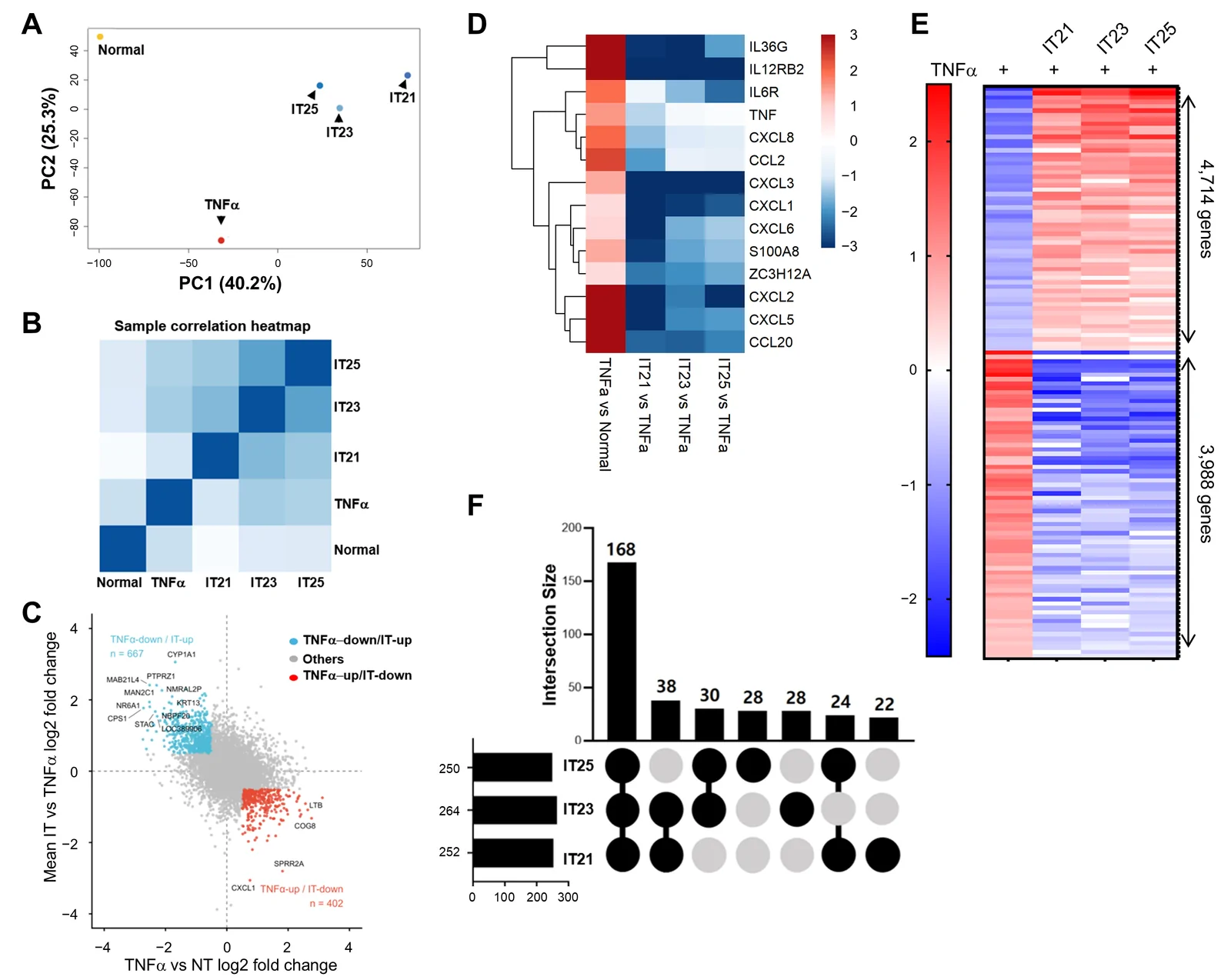
Exploratory transcriptomic profiling of candidate inflammation-associated expression patterns in TNFα-stimulated keratinocytes. (**A**) Principal component analysis (PCA) of transcriptomic profiles from untreated control (Normal), TNFα-stimulated (5 ng/mL), and TNFα plus IT21-, IT23-, or IT25-treated (5 μM) HaCaT keratinocytes. PC1 and PC2 account for 40.2% and 25.3% of the total variance, respectively. (**B**) Sample-to-sample correlation heatmap illustrating the relative similarity of global expression profiles across the experimental conditions. (**C**) Scatter plot comparing TNFα-associated log2 fold changes (TNFα vs. Normal) with mean IT-associated log2 fold changes (mean IT vs. TNFα). A total of 402 candidate genes showed a TNFα-up/IT-down expression pattern, whereas 667 showed a TNFα-down/IT-up pattern, based on an absolute log2 fold-change threshold of ≥0.5. (**D**) Heatmap showing expression patterns of representative inflammatory cytokine-related genes and CC/CXC chemokines, including *TNF*, *IL6R*, *IL36G*, *IL12RB2*, *CCL2*, *CCL20*, *CXCL1*, *CXCL2*, *CXCL3*, *CXCL5*, *CXCL6*, and *CXCL8*, across the experimental conditions. (**E**) Heatmap illustrating broader transcriptomic expression patterns associated with TNFα stimulation and treatment with IT21, IT23, or IT25. (**F**) UpSet plot showing the intersection of 168 candidate inflammatory genes identified across the three EGR1 inhibitor-treated conditions through leading-edge analysis of selected inflammation-associated Hallmark gene sets. RNA sequencing was performed as an exploratory, hypothesis-generating screen with one sample per experimental condition (n = 1) and without independent biological replication. Accordingly, PCA, sample-to-sample correlation, expression-pattern, enrichment, and leading-edge analyses shown here are descriptive observations of the five singleton transcriptomic profiles and were used for candidate prioritization rather than for statistical inference of differential expression or biological reproducibility.

Principal component analysis (PCA) and sample-to-sample correlation analysis were used to descriptively visualize the overall transcriptomic relationships among the five individual samples representing the control, TNFα, TNFα + IT21, TNFα + IT23, and TNFα + IT25 conditions (Figure 1A,B). Because each condition was represented by a single sample, these patterns are descriptive observations of the five individual transcriptomic profiles and should not be interpreted as evidence of biological reproducibility or replication.

To identify candidate genes showing IT-associated reversal of TNFα-induced transcriptional changes, genes altered by TNFα relative to the untreated control were first identified using the predefined expression-change criteria described in the Methods. Among these genes, those showing an opposite-direction change following treatment with IT21, IT23, and IT25 were subsequently selected. Application of these sequential filtering criteria identified 168 candidate genes commonly showing an IT-associated reversal pattern. Accordingly, these genes were considered exploratory candidates rather than statistically validated differentially expressed genes.

Because each condition was represented by a single sample, these transcriptomic profiles were interpreted descriptively and were not used for statistical inference regarding biological reproducibility or pathway-level effects. Candidate genes showing opposing expression patterns between TNFα stimulation and IT compound treatment were therefore prioritized using sequential fold-change-based criteria for subsequent biological validation. Collectively, this exploratory analysis was therefore used solely for candidate prioritization, with *CCL2* subsequently selected for independent biological and mechanistic validation.

### 2.2. Exploratory Multi-Compound Analysis Prioritizes Candidate Chemokines for Independent Validation

To prioritize candidate genes for subsequent investigation, we compared expression patterns observed in the three IT-treated singleton samples. Because IT21, IT23, and IT25 are structurally related compounds and each treatment condition was represented by a single sample, similarities among these profiles were used only for candidate prioritization and were not interpreted as evidence of biological reproducibility or independent validation. Genes showing TNFα-induced expression changes that were reversed in the IT-treated conditions were screened using an absolute log_2_ fold-change threshold of ≥0.5.

Comparison of TNFα-associated expression changes with the mean changes observed following IT treatment identified 402 candidate genes that were increased by TNFα and showed an opposite expression trend following IT treatment (TNFα-up/IT-down), whereas 667 genes decreased by TNFα showed an opposite trend following IT treatment (TNFα-down/IT-up) (Figure 1C). These gene groups were therefore considered exploratory candidates showing similar expression patterns among the three compound-treated profiles rather than statistically defined differentially expressed genes.

Inspection of inflammatory cytokine and chemokine transcripts revealed that several CC and CXC chemokines induced by TNFα showed lower expression in the IT21-, IT23-, and IT25-treated samples (Figure 1D,E), including *CCL2*, *CCL20*, *CXCL1*, *CXCL2*, *CXCL3*, *CXCL5*, *CXCL6*, and *CXCL8*. These observations identified these chemokines as exploratory candidates for further investigation but do not establish reproducible or EGR1-dependent regulation.

To prioritize candidates for subsequent biological and mechanistic validation, leading-edge genes (CORE = Yes) were extracted from four inflammation-associated Hallmark gene sets: TNFA SIGNALING VIA NFKB, INFLAMMATORY RESPONSE, INTERFERON GAMMA RESPONSE, and IL6-JAK-STAT3 SIGNALING. Intersection analysis identified 168 candidate genes represented across the three IT-treated conditions (Figure 1F and Supplementary Figure S1A ). Ranking these candidates according to their cumulative occurrence across the analyzed gene sets placed *CCL2* among the most recurrently represented genes, together with *CCL5*, *IL6*, and *RIPK2* (Supplementary Figure S1B). These analyses were used solely for exploratory candidate prioritization. KEGG pathway mapping further positioned *CCL2* within the TNF-associated inflammatory signaling and leukocyte-recruitment response identified in the exploratory analysis (Supplementary Figure S2).

Because direct EGR1-dependent transcriptional regulation of *CCL2* has been demonstrated in breast cancer cells [13], *CCL2* was selected as a representative chemokine to determine whether this established regulatory relationship also operates in inflammatory keratinocytes. Subsequent experiments therefore focused on independently validating *CCL2* regulation and examining its EGR1-dependent promoter regulation in the keratinocyte context, rather than establishing a previously unrecognized EGR1–*CCL2* regulatory axis.

### 2.3. Pharmacological and Genetic Inhibition of Egr1 Attenuates Tnfα-Induced Ccl2 Expression in Hacat Keratinocytes

**Figure 2:**
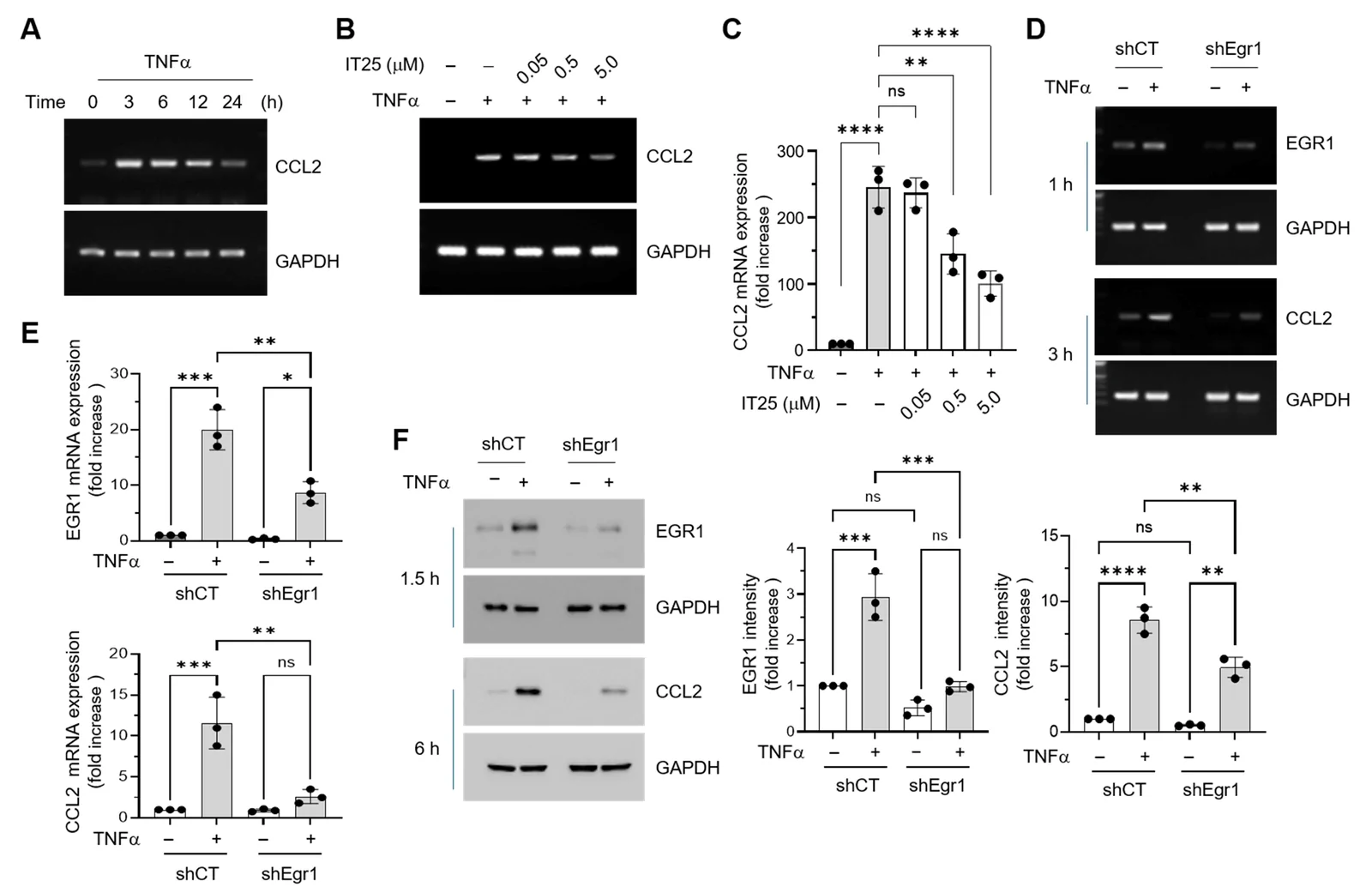
Pharmacological inhibition and genetic depletion of EGR1 attenuate TNFα-induced *CCL2* expression in HaCaT keratinocytes. (**A**) Time-course RT-PCR analysis of *CCL2* mRNA expression in HaCaT cells stimulated with 5 ng/mL TNFα for the indicated durations (0–24 h). (**B**) Semi-quantitative RT-PCR and (**C**) quantitative real-time PCR analysis showing dose-dependent suppression of *CCL2* transcripts by IT25 (0.05, 0.5, and 5.0 μM) under TNFα stimulation (5 ng/mL). (**D**) RT-PCR, (**E**) real-time qPCR, and (**F**) Western blot analysis of EGR1 and CCL2 protein expression in control (shCT) and EGR1-knockdown (shEgr1) cells following TNFα stimulation. EGR1 and CCL2 protein levels were quantified by densitometric analysis using ImageJ (version 1.52a) software and normalized to the corresponding GAPDH loading control. Data are presented as the mean ± SD from three independent experiments (n = 3). Homogeneity of variance was confirmed using the Brown–Forsythe test (*p* > 0.05). Statistical significance was determined by one-way ANOVA with Šidák’s multiple comparisons test (**C**) or two-way ANOVA with Tukey’s multiple comparisons test (**E**,**F**): * *p* < 0.05, ** *p* < 0.01, *** *p* < 0.001, **** *p* < 0.0001; ns, not significant.

To independently validate a representative candidate emerging from the exploratory transcriptomic screen, we examined the expression kinetics and EGR1 dependency of *CCL2* in HaCaT keratinocytes using independent biological experiments. Time-course RT-PCR showed that TNFα (5 ng/mL) rapidly induced *CCL2* mRNA expression, which increased between 3 and 6 h and remained elevated for up to 24 h (Figure 2A).

Among the three EGR1 inhibitors examined in the initial transcriptomic screen, IT25 was selected for subsequent validation based on its previously characterized EGR1-inhibitory and therapeutic effects against AD-like skin inflammation [12]. Treatment with increasing concentrations of IT25 (0.05, 0.5, and 5.0 μM) progressively reduced TNFα-induced *CCL2* mRNA expression, as assessed by semi-quantitative RT-PCR (Figure 2B). Quantitative real-time PCR using independent biological triplicates (n = 3) confirmed a concentration-dependent reduction in *CCL2* transcript levels, with the greatest suppression observed at 5.0 μM IT25 (*p* < 0.0001; Figure 2C).

To determine whether EGR1 contributes to TNFα-induced *CCL2* expression independently of pharmacological inhibition, HaCaT cells stably expressing non-targeting control shRNA (shCT) or EGR1-targeting shRNA (shEgr1) were examined. EGR1 knockdown was confirmed at both the mRNA and protein levels (Figure 2D, top panel; Figure 2F). TNFα stimulation increased *CCL2* mRNA expression by 11.59 ± 3.15-fold in shCT cells, whereas this induction was reduced to 2.90 ± 0.86-fold in shEgr1 cells (n = 3, *p* < 0.001; Figure 2D,E). Consistent with the mRNA findings, IT25 reduced CCL2 protein expression in TNFα-stimulated keratinocytes. Densitometric analysis of three independent experiments confirmed the changes in CCL2 and EGR1 protein levels after normalization to GAPDH (Figure 2F).

Together, these independent pharmacological and genetic experiments support the involvement of EGR1 in TNFα-induced *CCL2* expression in inflammatory keratinocytes and provide biological validation of *CCL2* as a representative candidate identified through the exploratory transcriptomic screen.

### 2.4. Egr1-Dependent Regulation of the Proximal Ccl2 Promoter in Keratinocytes

Because direct transcriptional regulation of *CCL2* by EGR1 has previously been demonstrated in non-cutaneous cellular contexts, we next examined whether this regulatory mechanism is also operative in TNFα-stimulated keratinocytes. A series of 5′-truncated human *CCL2* promoter-luciferase constructs, pCCL2-Luc(−987/+6), pCCL2-Luc(−500/+6), pCCL2-Luc(−250/+6), and pCCL2-Luc(−105/+6), was generated to localize the TNFα-responsive proximal promoter region (Supplementary Figure S3A). All deletion constructs retained responsiveness to TNFα, suggesting that a TNFα-responsive cis-regulatory element is contained within the proximal −105/+6-bp region. Sequence analysis of this region identified a GC-rich putative EGR1-binding site (EBS; 5′-CCGCCCTCT-3′) immediately upstream of the TATA box (Supplementary Figure S3B).

**Figure 3:**
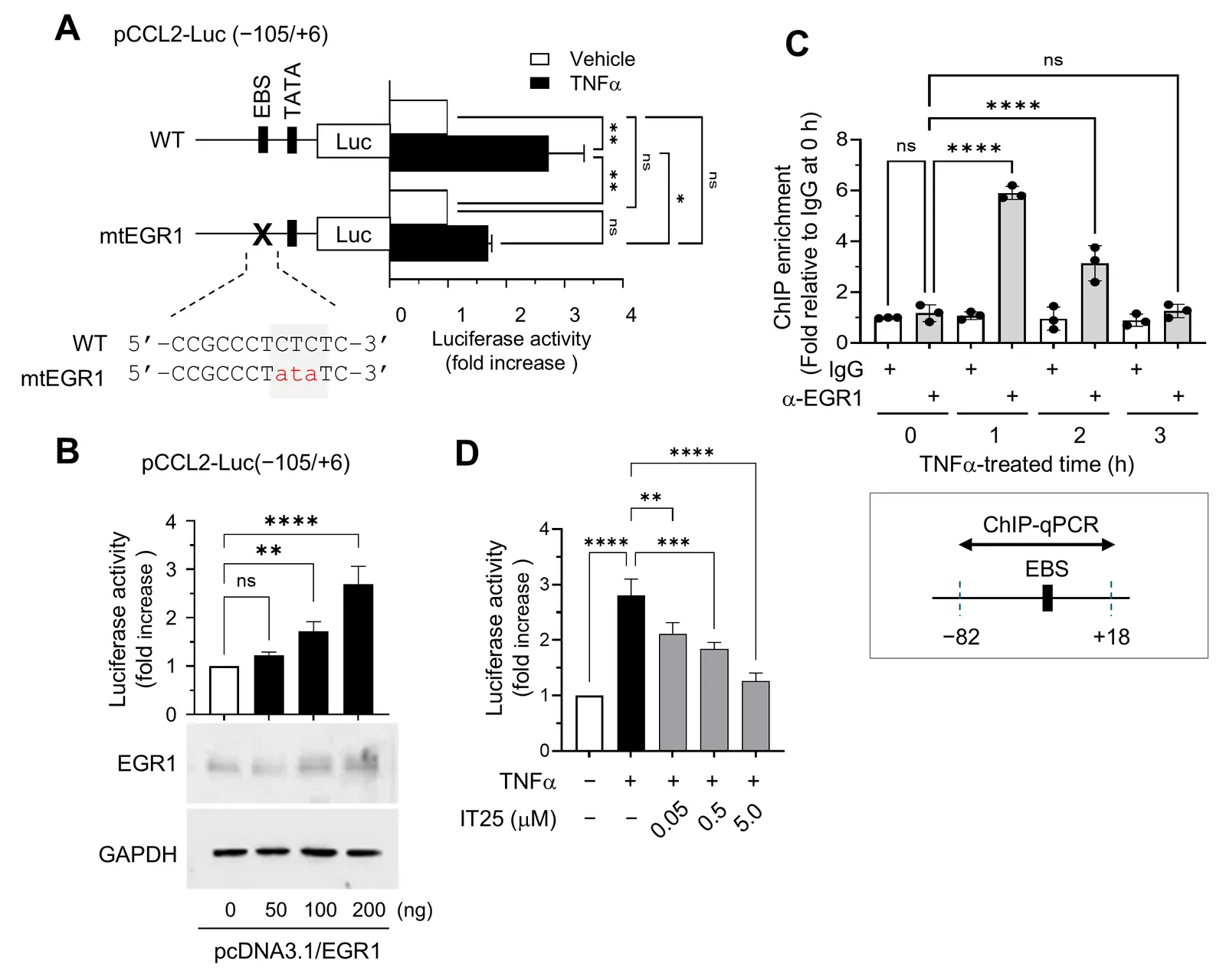
EGR1-dependent regulation of the proximal *CCL2* promoter in human keratinocytes. (**A**) Luciferase promoter–reporter assay in HaCaT cells transfected with the wild-type pCCL2-Luc(−105/+6) construct or the site-directed EBS mutant construct (mtEBS) following 8 h stimulation with 5 ng/mL TNFα. The mutated nucleotide is typed in red. (**B**) Luciferase reporter activity in resting HaCaT cells co-transfected with pCCL2-Luc(−105/+6) and increasing amounts of EGR1 expression plasmid (pcDNA3.1/EGR1: 0, 50, 100, 200 ng). (**C**) Chromatin immunoprecipitation (ChIP)-qPCR kinetics showing time-dependent recruitment of endogenous EGR1 to the proximal *CCL2* promoter (−82 to +18 bp) following TNFα treatment. ChIP enrichment was first calculated as % input and subsequently expressed as fold change relative to the IgG control at 0 h, which was set to 1. IgG immunoprecipitation was performed at each time point. (**D**) Dose-dependent suppression of TNFα-induced proximal *CCL2* promoter activity by IT25 (0, 0.05, 0.5, and 5.0 μM). Data are presented as the mean ± SD (n = 3). For (**A**), statistical significance was assessed by two-way ANOVA with promoter construct and TNFα treatment as factors, followed by Tukey’s multiple-comparisons test. A significant construct × treatment interaction was observed (F(1, 8) = 18.02, *p* = 0.0028). For (**B**,**D**), homogeneity of variance was assessed using the Brown–Forsythe test, and no significant heterogeneity was detected (*p* > 0.05). Statistical significance was assessed by one-way ANOVA followed by Dunnett’s multiple-comparisons test for (**B**) and Šídák’s multiple-comparisons test for (**D**). For (**C**), statistical significance was assessed by two-way ANOVA with TNFα treatment time and ChIP antibody as factors, followed by Dunnett’s multiple-comparisons test comparing α-EGR1 enrichment at each time point with α-EGR1 enrichment at 0 h. A significant time × antibody interaction was observed (F(3, 16) = 55.37, *p* < 0.0001). ns, not significant; ** *p* < 0.01; *** *p* < 0.001; **** *p* < 0.0001.

To assess the functional contribution of this EBS, site-directed mutagenesis was performed in the pCCL2-Luc(−105/+6) construct (Figure 3A). Basal promoter activity did not differ significantly between the WT and mtEBS constructs (adjusted *p* > 0.9999). Two-way ANOVA using promoter construct (WT versus mtEBS) and TNFα treatment as independent factors revealed a significant construct-by-treatment interaction (F(1, 8) = 18.02, *p* = 0.0028), indicating that the promoter response to TNFα differed between the WT and mtEBS constructs. When TNFα responsiveness was evaluated relative to the basal activity of each respective construct, TNFα increased WT promoter activity by approximately 2.73-fold (adjusted *p* = 0.0020), whereas the mtEBS construct showed a smaller increase of approximately 1.70-fold that did not reach statistical significance (adjusted *p* = 0.1164). Under TNFα-stimulated conditions, promoter activity was also significantly lower for mtEBS than for the WT construct (adjusted *p* = 0.0248). These findings indicate that mutation of the proximal EBS alters the TNFα responsiveness of the *CCL2* promoter without significantly affecting basal promoter activity. Together with the attenuation of TNFα-induced CCL2 expression following EGR1 knockdown and the TNFα-induced recruitment of endogenous EGR1 to the proximal CCL2 promoter, these findings support the involvement of EGR1 in TNFα-responsive CCL2 regulation.

We next examined whether increased EGR1 expression was sufficient to enhance *CCL2* promoter activity. Co-transfection of the proximal *CCL2* promoter construct with increasing amounts of an EGR1 expression plasmid resulted in a concentration-dependent increase in promoter activity in the absence of TNFα, accompanied by increased EGR1 protein expression (n = 3, *p* < 0.0001; Figure 3B).

Chromatin immunoprecipitation (ChIP)-qPCR analysis demonstrated transient recruitment of endogenous EGR1 to the proximal *CCL2* promoter following TNFα stimulation, with maximal enrichment observed at 1 h (Figure 3C). Two-way ANOVA with TNFα treatment time and ChIP antibody as factors revealed a significant time-by-antibody interaction (F(3, 16) = 55.37, *p* < 0.0001). EGR1 enrichment was significantly increased at 1 and 2 h relative to 0 h and returned toward basal levels by 3 h. In addition, IT25 treatment reduced TNFα-induced activity of the proximal *CCL2* promoter in a concentration-dependent manner (n = 3, *p* < 0.0001; Figure 3D).

Collectively, these findings confirm that the previously described EGR1-dependent regulation of *CCL2* is also operative in inflammatory keratinocytes and provide context-specific mechanistic validation linking pharmacological EGR1 inhibition to reduced *CCL2* transcription.

### 2.5. Histopathological Changes in Hdm-Challenged Skin Following Topical It25 Treatment

**Figure 4:**
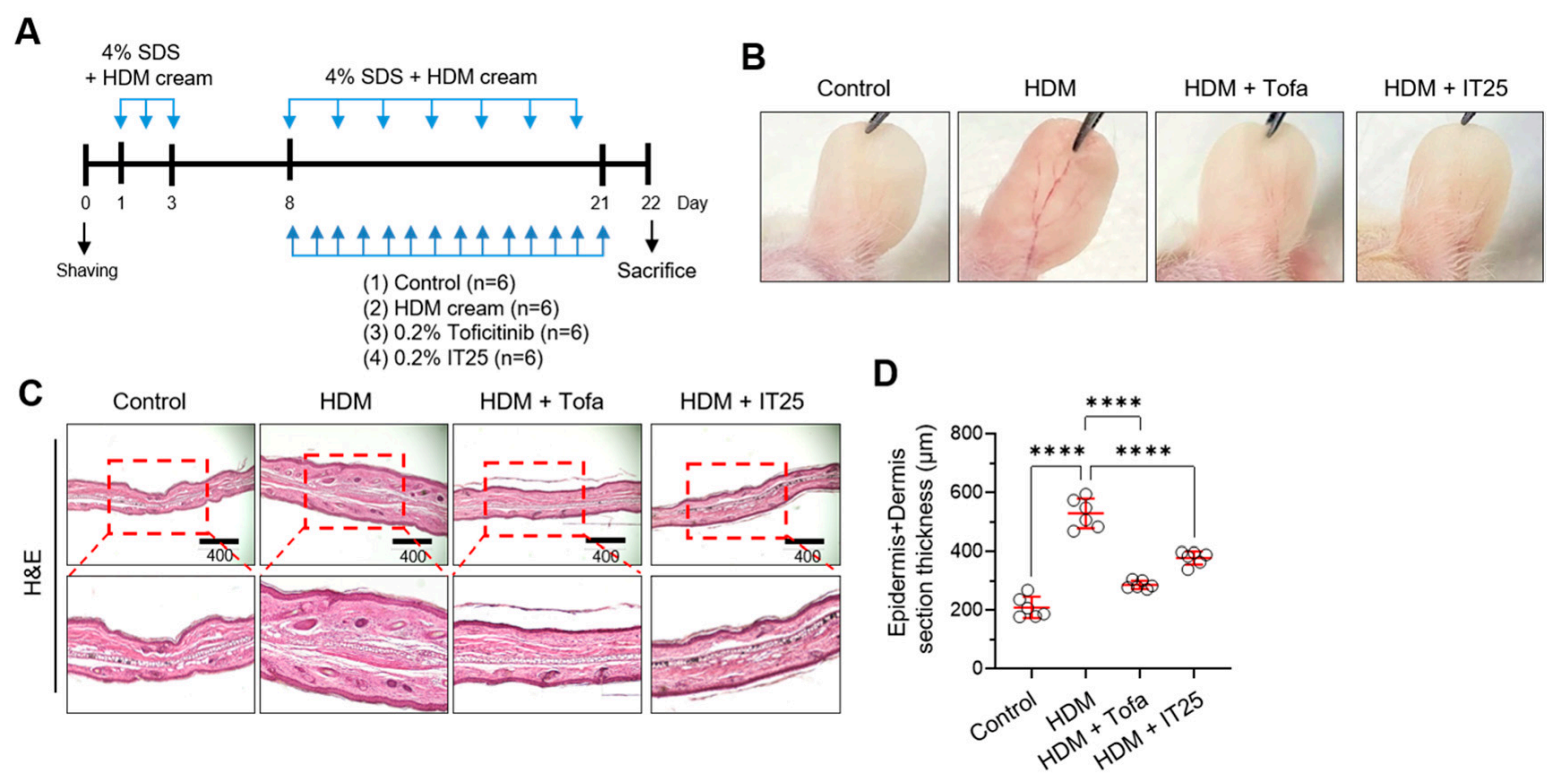
Topical administration of IT25 ameliorates house dust mite (HDM)-induced atopic dermatitis-like skin lesions in BALB/c mice. (**A**) Schematic of the experimental protocol. Before each HDM challenge, 4% SDS was applied to the ear skin for epidermal barrier disruption, followed 2–3 h later by topical application of HDM extract cream (50 mg/ear). HDM challenges were performed on days 1, 2, 3, 8, 10, 12, 14, 16, 18, and 20. IT25 or tofacitinib (0.2% in 2.5% Pluronic F-127; 50 μL/ear) was topically administered daily from days 8 to 21 and, on HDM challenge days, was applied 30 min before HDM. Mice were sacrificed on day 22 for tissue collection. (**B**) Representative macroscopic clinical photographs of ear skin lesions on day 22 across Control, HDM, HDM + Tofacitinib, and HDM + IT25 groups. (**C**) Representative H&E-stained ear sections collected on day 22 (scale bars = 400 μm). (**D**) Quantification of total histological tissue thickness (epidermis plus dermis) from H&E-stained ear sections using ImageJ. Data represent the mean ± SD (n = 6 mice per group). Statistical significance was assessed by one-way ANOVA followed by Šídák’s multiple-comparisons test for prespecified comparisons (Control vs. HDM, HDM vs. HDM + tofacitinib, and HDM vs. HDM + IT25). **** *p* < 0.0001.

House dust mite (HDM) is a major environmental allergen that induces atopic dermatitis (AD)-like cutaneous inflammation primarily through Toll-like receptor (TLR)-mediated signaling [14]. Among the downstream inflammatory mediators, TNFα functions as a key upstream cytokine driving the development of HDM-induced skin lesions [15,16]. To characterize the inflammatory skin context for evaluating the in vivo effects of IT25, an HDM-induced AD-like skin inflammation model was established in BALB/c mice (Figure 4A). In our previous preliminary safety baseline test for topical IT25, we evaluated its cytotoxicity in HaCaT keratinocytes and local tolerability in normal mouse skin [12]. IT25 showed no detectable cytotoxicity at concentrations up to 100 μM, and topical application at concentrations up to 2% caused no apparent adverse effects or gross skin abnormalities in normal mice. Before each HDM challenge, 4% SDS was applied to disrupt the epidermal barrier. Mice received topical vehicle, IT25 (0.2%), or the JAK inhibitor tofacitinib (0.2%) as a reference treatment [17].

Repeated HDM challenge produced visible skin changes, including erythema, excoriation, and crusting (Figure 4B). Because a prespecified dermatitis severity scoring system was not applied, the gross photographs are presented as representative qualitative images only. Histopathological examination of H&E-stained ear sections collected on day 22 showed epidermal hyperplasia, acanthosis, hyperkeratosis, and dermal edema following HDM challenge (Figure 4C). Quantification of total histological tissue thickness (epidermis plus dermis) showed a significant increase in HDM-challenged mice and lower values in the IT25- and tofacitinib-treated groups (Figure 4D).

### 2.6. Epidermal Ccl2 Expression and Inflammatory Cell Infiltration Following Topical It25 Treatment

**Figure 5:**
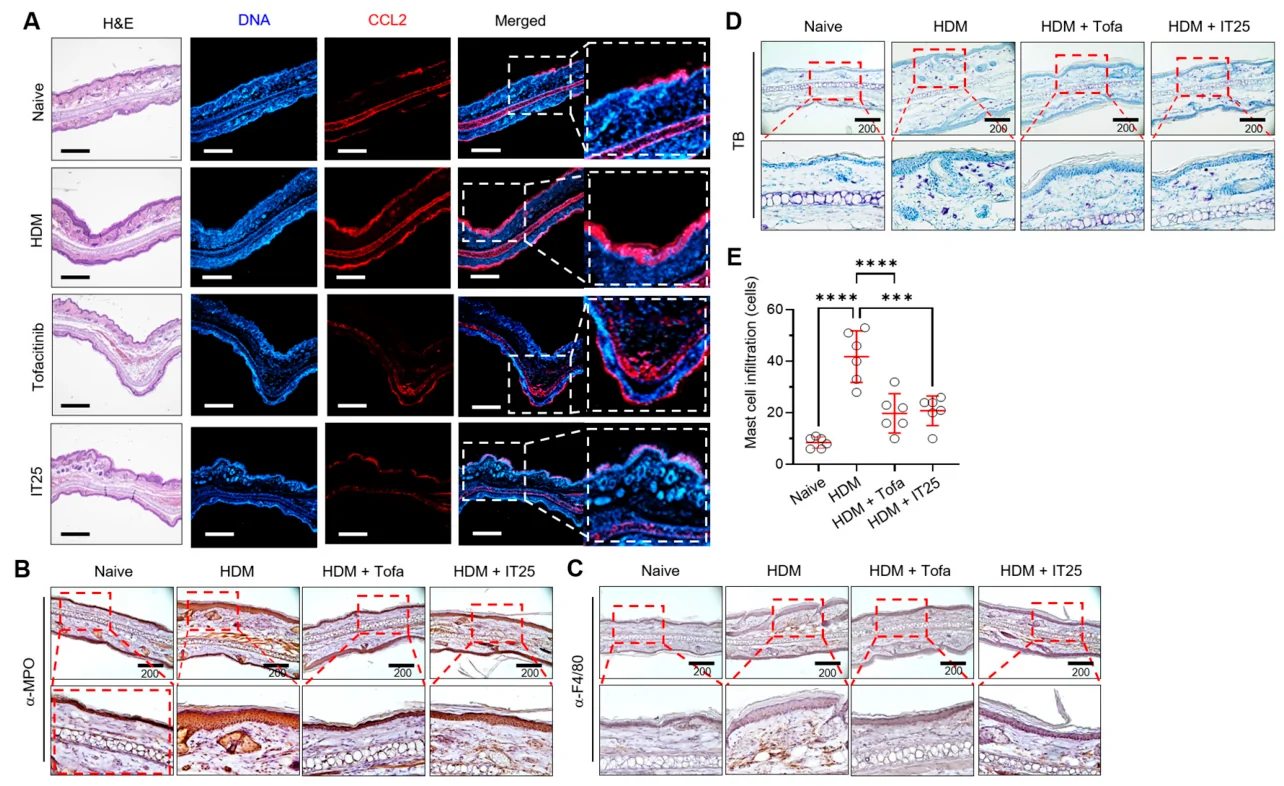
Topical IT25 attenuates epidermal CCL2 expression and multi-lineage leukocyte infiltration in HDM-sensitized murine skin. (**A**) Representative immunofluorescence images of ear cross-sections stained for CCL2 (red) and counterstained with Hoechst 33258 for nuclei (blue). Insets show higher-magnification views of the epidermis (scale bars = 400 μm). (**B**,**C**) Immunohistochemical staining of ear sections showing dermal infiltration of MPO-positive (**B**) and F4/80-positive cells (**C**) (scale bars = 200 μm; lower panels display enlarged views of dashed boxes). Representative images of CCL2, MPO, and F4/80 staining in ear skin are shown. These images are presented for qualitative comparison only and were not subjected to animal-level quantitative analysis. (**D**) Toluidine blue (TB) histological staining of dermal mast cells (scale bars = 200 μm) and (**E**) quantitative cell density analysis of infiltrating mast cells. Data represent the mean ± SD (n = 6 per group). Statistical significance: *** *p* < 0.001, **** *p* < 0.0001 (by one-way ANOVA with Dunnett’s multiple comparisons test).

To determine whether the inhibitory effect of IT25 on CCL2 expression observed in inflammatory keratinocytes was also reflected in vivo, we examined epidermal CCL2 expression in HDM-challenged skin. Representative immunofluorescence images showed lower epidermal CCL2 immunoreactivity in IT25-treated mice than in HDM-challenged mice (Figure 5A). Because these images were not subjected to animal-level quantitative analysis, this observation is presented qualitatively and does not establish quantitative suppression of CCL2 expression in vivo.

Because HDM-induced skin inflammation is characterized by extensive infiltration of innate immune cells [18], including macrophages and mast cells, CCL2 is a crucial chemokine involved in the recruitment of monocytes and other leukocyte subsets [19]. MPO- and F4/80-positive cells (Figure 5B,C) appeared less prominent in IT25-treated skin. Because these immunohistochemical images were not suitable for reliable animal-level quantitative analysis, these observations are presented qualitatively and should not be interpreted as quantitative evidence of reduced inflammatory-cell infiltration. Toluidine blue staining further demonstrated increased mast cell accumulation in HDM-challenged skin, which was significantly reduced by IT25 treatment (n = 6, *p* < 0.001; Figure 5D,E). Together, these findings show that topical IT25 treatment was associated with qualitatively less prominent epidermal CCL2 immunoreactivity and MPO- and F4/80-positive staining, together with a quantitatively demonstrated reduction in mast cell accumulation. These observations are consistent with attenuation of the inflammatory tissue environment but do not establish that suppression of CCL2 or other individual chemokines directly accounts for the observed inflammatory changes.

Collectively, these in vivo findings demonstrate that topical IT25 suppresses epidermal chemokine production and limits the recruitment of multiple inflammatory cell lineages, leading to comprehensive alleviation of allergic cutaneous inflammation.

### 2.7. Validation of Chemokine Expression in an Independent Human Ad Transcriptomic Dataset

**Figure 6:**
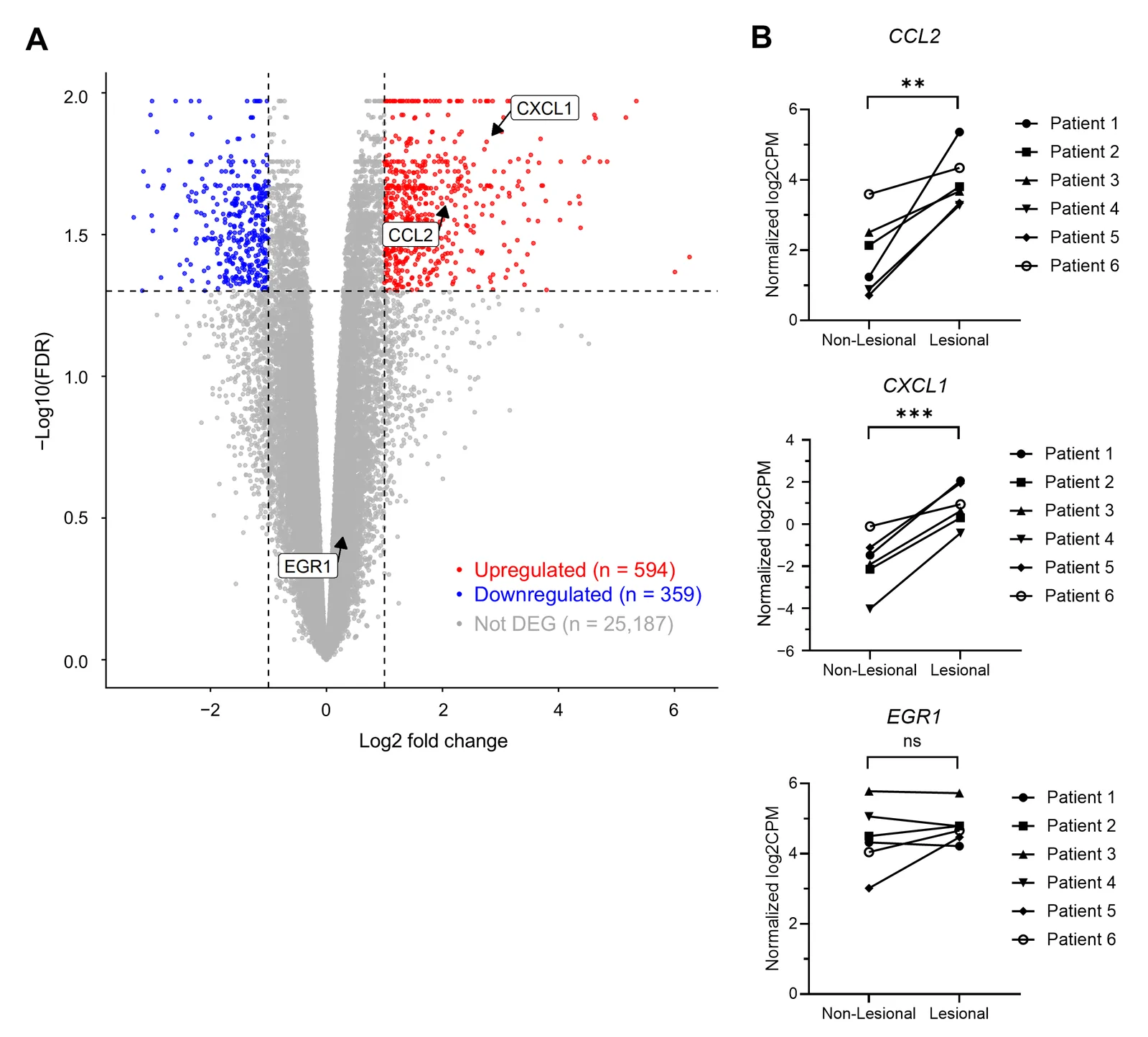
Expression of *CCL2*, *CXCL1*, and *EGR1* in human atopic dermatitis skin. (**A**) Differentially expressed genes (DEGs) between paired lesional and non-lesional skin samples from six patients with atopic dermatitis (GSE140227) were visualized in a volcano plot. DEGs were defined as |log2FC| ≥ 1 and false discovery rate (FDR) ≤ 0.05. *CCL2*, *CXCL1*, and *EGR1* are indicated. (**B**) Paired expression plots showing normalized log2 counts per million (log2CPM) values of *CCL2*, *CXCL1*, and *EGR1*. Each line connects non-lesional and lesional skin samples from the same patient (n = 6 paired samples). Statistical significance was assessed using a two-tailed paired *t*-test. ** *p* < 0.01, *** *p* < 0.001; ns, not significant.

To examine the clinical relevance of the chemokine responses identified in the present study, we analyzed the publicly available RNA-seq dataset GSE140227, comprising paired lesional and non-lesional skin samples from six patients with AD. Differential-expression analysis using a paired design showed that *CCL2* expression was significantly increased in lesional skin (log2FC = 2.08, FDR = 0.023). *CXCL1*, another chemokine identified in our transcriptomic analysis, was also significantly increased (log2FC = 2.76, FDR = 0.015), whereas *EGR1* transcript levels were not significantly different between paired non-lesional and lesional skin samples (log2FC = 0.31, FDR = 0.345) (Figure 6A). Consistent with the differential-expression analysis, paired comparison of normalized log2CPM values showed significantly higher *CCL2* and *CXCL1* expression in lesional skin, whereas *EGR1* showed no significant difference (Figure 6B).

## 3. Discussion

Persistent activation of inflammatory transcriptional programs in epidermal keratinocytes contributes to chronic cutaneous inflammatory disorders, including atopic dermatitis and psoriasis [1,2,3]. Because multiple cytokines and chemokines can exert overlapping or compensatory functions, targeting individual downstream mediators may provide incomplete control of complex inflammatory responses [3,5]. Pharmacological targeting of upstream transcriptional regulators capable of influencing multiple inflammatory mediators may therefore represent an alternative strategy for modulating cutaneous inflammation [6].

In the present study, we explored whether pharmacological inhibition of EGR1 DNA-binding activity influences broader inflammatory transcriptional responses in TNFα-stimulated keratinocytes. An exploratory transcriptomic screen using three structurally related EGR1 inhibitors identified candidate expression patterns in multiple inflammation-associated genes, including several leukocyte-recruiting chemokines. Because the RNA-seq screen was performed without independent biological replicates for each sequencing condition, these transcriptomic observations were used for hypothesis generation and candidate prioritization rather than as definitive evidence of an EGR1-dependent transcriptional network. *CCL2* was subsequently selected as a representative candidate for independent biological and mechanistic validation. Pharmacological inhibition and genetic silencing of EGR1 both attenuated TNFα-induced CCL2 expression. Promoter and ChIP analyses further confirmed that the previously reported EGR1-dependent transcriptional regulation of *CCL2* [13] is also operative in inflammatory keratinocytes. In vivo, topical IT25 attenuated HDM-induced skin inflammation, accompanied by reduced CCL2 expression and infiltration of several inflammatory cell populations.

### 3.1. Egr1-Dependent Regulation of Ccl2 in the Context of Epidermal Inflammation

Direct transcriptional regulation of *CCL2* (MCP-1) by EGR1 has previously been demonstrated in non-cutaneous disease contexts [13]. EGR1 has also been implicated in the transcriptional regulation of several other inflammatory mediators, including *IL6* and *IL8*. Our previous studies demonstrated EGR1-dependent regulation of individual inflammatory genes in keratinocytes and described the development and initial pharmacological characterization of IT21, IT23, and IT25, including their anti-inflammatory and anti-AD effects [11,12]. Thus, the present study extends our previous work rather than establishing these compounds or their anti-dermatitis activity de novo. Here, these compounds were used as pharmacological tools in independently performed experiments to explore downstream inflammatory transcriptional responses. The exploratory transcriptomic screen identified expression patterns involving several leukocyte-recruiting chemokines, including CCL2, CCL20, CXCL1, CXCL2, CXCL3, CXCL5, CXCL6, and CXCL8. Because the three compound-treated samples do not constitute biological replicates, these observations were used for candidate prioritization rather than as evidence of reproducible or direct EGR1-dependent regulation. Accordingly, the other chemokines identified in the exploratory RNA-seq analysis should be regarded as candidate responses requiring independent validation, whereas *CCL2* was subsequently selected for independent biological and mechanistic validation. EGR1-dependent regulation of *CCL2* has previously been reported in breast cancer cells, and the anti-inflammatory activity of IT25 in experimental AD has also been described in our previous study. The present findings therefore do not establish *CCL2* as a previously unrecognized EGR1 target or the anti-inflammatory activity of IT25 as a new pharmacological observation. Rather, they extend these prior findings by demonstrating a context-specific EGR1–*CCL2* transcriptional mechanism in inflammatory keratinocytes, supported by pharmacological and genetic perturbation, proximal promoter analysis, EGR1-binding-site mutation, and endogenous EGR1 recruitment assessed by ChIP-qPCR. All experimental data presented here were independently generated for the present study and were not reused from our previous publication [12].

Independent experiments demonstrated that IT25 reduced TNFα-induced *CCL2* expression in a concentration-dependent manner and that genetic depletion of EGR1 similarly attenuated *CCL2* expression. These complementary pharmacological and genetic observations support EGR1 involvement in *CCL2* regulation in inflammatory keratinocytes.

Promoter deletion analysis localized TNFα responsiveness to the proximal *CCL2* promoter, and mutation of the putative EGR1-binding sequence markedly impaired promoter activation. Ectopic EGR1 expression enhanced *CCL2* promoter activity, while ChIP-qPCR demonstrated increased endogenous EGR1 occupancy at the proximal *CCL2* promoter following TNFα stimulation. IT25 additionally reduced TNFα-induced *CCL2* promoter activity. Mutation of the putative EGR1-binding sequence significantly altered the TNFα responsiveness of the *CCL2* promoter but also markedly reduced basal promoter activity. Because this sequence lies within a GC-rich proximal promoter region, the mutational effect may reflect disruption of basal promoter architecture or binding of other GC-rich-sequence-recognizing transcription factors in addition to EGR1. Thus, the promoter-mutagenesis data support the involvement of this proximal region in TNFα-responsive transcription but do not independently establish EGR1 specificity. Additional approaches, such as EGR1 knockdown/rescue in the promoter assay or alternative mutations that preserve local promoter architecture, will be required to establish sequence-specific EGR1 regulation more definitively. Together, these findings provide context-specific confirmation that EGR1-dependent *CCL2* transcription occurs in inflammatory keratinocytes, while the regulation of the other chemokines identified in the exploratory screen remains to be independently validated.

The in vivo findings further support the biological relevance of pharmacological EGR1 inhibition in cutaneous inflammation. Topical IT25 reduced HDM-induced ear thickening and histopathological changes and was associated with decreased epidermal CCL2 expression. IT25 treatment was also associated with less prominent F4/80- and MPO-positive staining and a significant reduction in mast cell accumulation. Notably, IT25 showed no detectable cytotoxicity in keratinocytes at concentrations up to 100 μM and was well tolerated following topical application at concentrations up to 2% in normal mouse skin, supporting a preliminary safety margin for topical administration. However, comprehensive dermal toxicity studies will be required to establish its long-term safety profile. These findings are consistent with an overall attenuation of the inflammatory tissue environment following topical IT25 treatment. However, because individual chemokines were not selectively manipulated in vivo, the reduction in inflammatory cell infiltration cannot be directly attributed to suppression of CCL2 or any specific chemokine identified in the transcriptomic screen. Rather, the in vivo findings provide qualitative evidence of attenuated inflammatory features in IT25-treated skin, including less prominent CCL2 immunoreactivity and accumulation of MPO- and F4/80-positive cells, without establishing quantitative changes in these markers or a causal role for CCL2 or the other chemokines identified in the exploratory transcriptomic screen.

Importantly, the present in vivo experiments do not directly demonstrate EGR1 target engagement or inhibition of EGR1 DNA-binding activity in IT25-treated skin. Therefore, the observed anti-inflammatory effects cannot be attributed exclusively to EGR1 inhibition, and the qualitative changes in CCL2 immunoreactivity and inflammatory-cell accumulation may, at least in part, reflect a secondary consequence of the overall attenuation of skin inflammation. Moreover, because the selectivity of IT25 against other potential molecular targets was not evaluated in the present in vivo experiments, EGR1-independent or off-target effects cannot be excluded. Direct assessment of EGR1 target engagement and downstream transcriptional activity in treated skin will therefore be necessary to establish the mechanistic basis of the in vivo pharmacological effects of IT25.

The clinical relevance of EGR1 dysregulation in AD is supported by our previous demonstration of increased EGR1 expression in lesional skin biopsies from patients with AD compared with normal human skin [20]. In the independent human AD transcriptomic dataset GSE140227 analyzed in the present study, *CCL2* and *CXCL1* were significantly increased in lesional skin compared with paired non-lesional skin, whereas *EGR1* transcript levels were not significantly altered (Figure 6). This finding indicates that steady-state *EGR1* mRNA abundance does not necessarily parallel chemokine expression in human lesions and underscores the need for caution in extrapolating the experimentally defined EGR1-dependent mechanism directly to human AD. Nevertheless, together with our previous observation of increased EGR1 expression in AD lesional skin, these findings support further investigation of the relationship between EGR1 activity and chemokine dysregulation in human inflammatory skin disease. Collectively, these human observations support the clinical relevance of the EGR1-associated chemokine responses identified in our experimental models, although they do not by themselves establish a direct causal relationship between EGR1 and chemokine expression in human AD lesions.

An additional limitation is that the mechanistic experiments were performed primarily in the immortalized HaCaT keratinocyte line. Although this model provides a reproducible system for dissecting transcriptional regulation, immortalized keratinocytes may not fully recapitulate the responses of primary human epidermal keratinocytes or the multicellular architecture of human skin. Validation of the EGR1–CCL2 regulatory mechanism in primary human keratinocytes and organotypic skin models will therefore be important to establish its broader physiological and translational relevance.

### 3.2. Study Limitations and Future Perspectives

Several limitations of this study should be acknowledged. First, the RNA-seq experiment was conducted as an exploratory screen with one sample per experimental condition. The absence of independent biological replicates precludes robust estimation of biological variability and limits statistical inference from the sequencing dataset. Accordingly, the transcriptomic data were used primarily to identify candidate expression patterns rather than to establish statistically definitive differential expression. For candidate prioritization, we focused on expression patterns observed across the three structurally related compounds. However, because these compounds share structural features, concordance among them may reflect shared pharmacological or off-target effects and does not substitute for independent biological replication. We therefore selected *CCL2* as a representative candidate for subsequent independent biological and mechanistic validation. Future transcriptomic studies with adequate biological replication, together with targeted validation of additional chemokines, will be required to determine the broader inflammatory transcriptional responses associated with EGR1-targeting compounds.

Second, although *CCL2* was independently validated as an EGR1-regulated chemokine in keratinocytes, CCL2 protein was assessed in cell lysates, and the present study therefore does not directly establish whether EGR1 inhibition alters extracellular CCL2 secretion. In addition, the functional contribution of individual chemokines to the anti-AD effects of IT25 was not directly determined. Chemokine-specific rescue or neutralization experiments were not performed, and therefore the relative contribution of *CCL2* or other chemokines to particular inflammatory cell populations or histopathological outcomes remains unresolved. Future studies employing chemokine-specific genetic or pharmacological approaches will be required to establish these causal relationships. A further limitation is that the CCL2, MPO, and F4/80 immunofluorescence findings were assessed qualitatively because the acquired images did not permit reliable animal-level retrospective quantification; therefore, these observations should be interpreted as supportive rather than quantitative evidence of the in vivo anti-inflammatory effects of IT25.

Third, keratinocytes represent an important source of inflammatory mediators in the epidermis, but dermal fibroblasts, endothelial cells, resident immune cells, and infiltrating leukocytes may also express EGR1 and contribute to the local chemokine milieu. Consequently, the cellular origin of the in vivo effects of IT25 cannot be attributed exclusively to keratinocytes. Single-cell transcriptomic approaches and cell-type-specific genetic models should help define the relative contribution of EGR1 signaling in individual cutaneous cell populations.

Fourth, an additional limitation is that the molecular selectivity of IT21, IT23, and IT25 for EGR1 has not yet been established. Because EGR1 belongs to the C2H2 zinc-finger transcription factor family, structurally related transcription factors, including EGR2/3, SP1, and KLF family members, may represent potential off-targets. Thus, although the present pharmacological, genetic, and promoter-occupancy data support modulation of EGR1-dependent transcriptional activity, they do not establish selective molecular targeting of EGR1 by these compounds. This limitation is particularly relevant to the interpretation of the in vivo findings, because the present animal experiments did not include an *Egr1*-deficient comparison or a structurally unrelated EGR1 inhibitor. Therefore, the attenuation of allergic skin inflammation following topical IT25 treatment should be regarded as pharmacological evidence consistent with EGR1 pathway modulation rather than as definitive evidence that the in vivo phenotype is caused specifically by EGR1 inhibition. Comparative biochemical and cellular profiling against related C2H2 zinc-finger transcription factors, together with genetic or orthogonal pharmacological validation, will be required to establish molecular selectivity and EGR1-specific causality in vivo.

Fifth, the in vivo experiments were conducted exclusively in male BALB/c mice. Given the potential influence of sex on AD-related inflammatory responses, the present findings cannot be assumed to generalize equivalently to females. The current study was not designed to evaluate sex as a biological variable. Future studies including both male and female animals will therefore be necessary to determine whether the response to topical IT25 and the associated inflammatory changes are influenced by sex. In addition, in vivo CCL2 protein expression was assessed by immunofluorescence rather than by quantitative biochemical methods such as ELISA or immunoblotting of skin lysates. The immunofluorescence findings should therefore be interpreted as supportive histological evidence of the relative CCL2 staining pattern rather than as quantitative evidence of changes in CCL2 protein abundance. Future studies incorporating quantitative protein measurements will be required to confirm these in vivo observations.

IT25 was evaluated at only a single topical concentration (0.2%) in the present in vivo study. Consequently, these findings do not establish a dose–response relationship, minimally effective dose, or optimal topical dose for IT25. Future studies incorporating multiple concentrations and exposure-response analyses will be necessary to define its therapeutic dose range and translational potential.

Sixth, histological image acquisition and analysis were performed without blinding to treatment-group allocation, which may have introduced observer bias into the assessment of histological outcomes. Future studies incorporating blinded acquisition and quantitative analysis will be required to minimize this potential source of bias.

Finally, the exploratory pathway analysis indicated changes in gene sets associated with chromatin regulation following IT25 treatment. Whether these changes represent downstream consequences of altered EGR1 activity or additional pharmacological effects of IT25 remains unknown. Further biochemical and chromatin-based studies are needed to address this possibility.

## 4. Materials and Methods

### 4.1. Reagents and Small-Molecule Inhibitors

Recombinant human TNFα was purchased from ProSpec-Tany TechnoGene, Ltd. (Ness-Ziona, Israel). Biostir AD cream containing house dust mite (Dermatophagoides farinae) extract was obtained from Central Lab Animal Inc. (Seoul, Republic of Korea). Tofacitinib citrate was purchased from MedChemExpress (Monmouth Junction, NJ, USA). The small-molecule compounds IT21, IT23, and IT25, previously characterized as inhibitors of EGR1 DNA-binding activity, were synthesized as described previously [12]. Chemical compounds were dissolved in molecular biology-grade dimethyl sulfoxide (DMSO; Sigma-Aldrich, St. Louis, MO, USA) as 10 mM stock solutions and stored at −20 °C.

### 4.2. Cell Culture and Shrna-Mediated Stable Knockdown

Human immortalized keratinocyte HaCaT cells (Cell Lines Service, Eppelheim, Germany) were cultured in Dulbecco’s Modified Eagle Medium (DMEM; Welgene, Gyeongsan, Republic of Korea) supplemented with 10% heat-inactivated fetal bovine serum (FBS; HyClone, Logan, UT, USA), 100 units/mL penicillin, and 100 ug/mL streptomycin (Sigma-Aldrich) at 37 °C in a humidified 5% CO_2_ atmosphere.

To generate stable EGR1-knockdown cells, HaCaT cells were transduced with lentiviral particles expressing human EGR1-targeting short hairpin RNA (shEgr1) or non-targeting scrambled control shRNA (shCT) in the presence of 8 μg/mL polybrene (Sigma-Aldrich) as described previously [11,12]. Transduced cells were selected and maintained in culture medium containing 2 μg/mL puromycin (Sigma-Aldrich). Knockdown efficiency was verified by RT-PCR, quantitative real-time PCR, and Western blot analysis.

### 4.3. RNA Sequencing (Rna-Seq) and Exploratory Bioinformatic Analysis

HaCaT cells were pretreated with vehicle (0.1% DMSO) or 5 μM IT21, IT23, or IT25 for 30 min, followed by stimulation with TNFα (5 ng/mL) for 6 h. Total RNA was extracted using NucleoZOL reagent (MACHEREY-NAGEL, Düren, Germany) according to the manufacturer’s instructions. RNA quality and concentration were assessed using an Agilent 2100 Bioanalyzer (Agilent Technologies, Santa Clara, CA, USA). QuantSeq 3′ mRNA-seq library preparation and high-throughput sequencing were performed by e-Biogen (Seoul, Republic of Korea).

RNA sequencing was conducted as an exploratory, hypothesis-generating screen with one sample per experimental condition and without independent biological replication. The experiment was therefore not designed for statistical inference of differential expression or estimation of biological variability. Accordingly, the transcriptomic data were interpreted descriptively and used primarily to identify candidate expression patterns for subsequent independent investigation.

Transcript-level expression values were normalized, and genes showing expression changes following TNFα stimulation that exhibited an opposite expression trend following IT treatment were screened using an absolute log_2_ fold-change (|log_2_FC|) threshold of ≥0.5. Expression patterns observed across the IT21-, IT23-, and IT25-treated singleton samples were compared for exploratory candidate prioritization. Because these compounds are structurally related and each treatment condition was represented by a single sample, similarity among the compound-treated profiles was not considered evidence of biological replication, reproducibility, or EGR1-dependent regulation. Unsupervised principal component analysis (PCA), hierarchical clustering, and sample-to-sample correlation analyses were performed using R software (v4.2.2).

Because independent biological replicates were not included in the RNA-seq screening experiment, statistical measures derived from the sequencing dataset were interpreted descriptively and were not used as definitive evidence of differential expression. Selected candidates emerging from the exploratory transcriptomic analysis were subsequently evaluated in independent biological experiments using targeted molecular and biochemical assays, including quantitative real-time PCR, Western blotting, promoter–reporter assays, and ChIP-qPCR. The biological relevance of pharmacological EGR1 inhibition was further examined in an HDM-induced murine model of cutaneous inflammation.

### 4.4. RNA Extraction, Rt-Pcr, and Quantitative Real-Time Pcr (Qpcr)

Total RNA was extracted from HaCaT cells using NucleoZOL reagent. Complementary DNA (cDNA) was synthesized from 1 ug of total RNA using the HiSense cDNA Synthesis Master Mix (CellSafe, Yongin, Republic of Korea) according to the manufacturer’s instructions.

For semi-quantitative RT-PCR, target genes were amplified using Hot-start Taq Polymerase (Enzynomics, Daejeon, Republic of Korea). Amplified products were resolved on 2% agarose gels containing nucleic acid stain and visualized using a GD-1000 Gel Documentation System (Axygen, Union City, CA, USA). Quantitative real-time qPCR was performed on a CFX Connect Real-Time PCR Detection System (Bio-Rad Laboratories, Hercules, CA, USA) using SYBR Green I (MedChemExpress). Cycling conditions were: 95 °C for 10 min, followed by 40 cycles of 95 °C for 15 s, 58–60 °C for 20 s, and 72 °C for 20 s. Relative expression levels were normalized against GAPDH using the comparative Delta-Delta Cq method. Primer sequences are listed in Supplementary Table S1.

### 4.5. Western Blot Analysis

Cells were washed twice with ice-cold PBS and lysed in RIPA buffer (50 mM Tris-HCl pH 7.5, 150 mM NaCl, 1% Nonidet P-40, 0.5% sodium deoxycholate, 0.1% SDS) supplemented with protease and phosphatase inhibitor cocktails (Roche, Mannheim, Germany). Clarified protein lysates (20–30 μg) were resolved by 10–12% SDS-PAGE and electrotransferred onto nitrocellulose membranes (Bio-Rad).

Membranes were blocked in 5% non-fat dry milk in TBST for 1 h at room temperature and incubated overnight at 4 °C with primary antibodies against EGR1 (1:1000; Cell Signaling Technology, Danvers, MA, USA), CCL2 (1:1000; R&D Systems, Minneapolis, MN, USA), and GAPDH (1:5000; Santa Cruz Biotechnology, Dallas, TX, USA). After washing with TBST, membranes were incubated with HRP-conjugated secondary antibodies (1:5000; Thermo Fisher Scientific, Waltham, MA, USA) for 1 h. Immunoreactive bands were detected using SuperSignal West Pico Plus Chemiluminescent Substrate (Thermo Fisher Scientific) and quantified using ImageJ software (NIH, Bethesda, MD, USA). Antibodies used in this study are listed in Supplementary Table S2.

### 4.6. Plasmid Construction, Site-Directed Mutagenesis, and Luciferase Reporter Assays

Human *CCL2* promoter fragments spanning −987/+6, −500/+6, −250/+6, and −105/+6 bp were amplified from human genomic DNA and cloned into the pGL4.17(luc2/Neo) luciferase reporter vector (Promega, Madison, WI, USA). The mutant reporter pCCL2-Luc(−105/+6)mtEBS was generated by introducing substitutions within the core EGR1-binding sequence (EBS; 5′-CCGCCCTCT-3′) in the proximal promoter. The corresponding 12-bp sequence was changed from 5′-CCGCCCTCTCTC-3′ to 5′-CCGCCCTataTC-3′ using the EZchange Site-Directed Mutagenesis Kit (Enzynomics). Constructs were verified by Sanger sequencing.

HaCaT cells seeded in 12-well plates were transfected with 0.2 μg of reporter plasmids using Lipofectamine 2000 (Invitrogen, Carlsbad, CA, USA). For overexpression studies, cells were co-transfected with increasing amounts of pcDNA3.1/EGR1 (0, 50, 100, 200 ng). After 48 h, cells were treated with TNFα (5 ng/mL) in the presence or absence of IT25 for 8–12 h. Luciferase activity was measured using the Dual-Glo Luciferase Assay System (Promega) on a Centro LB960 microplate luminometer (Berthold Technologies, Bad Wildbad, Germany) and normalized to total protein content.

### 4.7. Chromatin Immunoprecipitation (Chip)-Qpcr Assay

ChIP assays were performed using the ChIP Assay Kit (Millipore, Billerica, MA, USA). HaCaT cells were cross-linked with 1% formaldehyde solution containing 0.1 M NaCl, 1 mM EDTA, and 50 mM HEPES pH 8.0 for 10 min at 25 °C, quenched with 125 mM glycine, and lysed in SDS lysis buffer. Chromatin was sheared to an average fragment size of 200–500 bp using an ultrasonic sonicator (Bioruptor, Diagenode, Liège, Belgium).

Soluble chromatin was immunoprecipitated overnight at 4 °C with 2 μg of anti-EGR1 antibody (Cell Signaling Technology) or normal rabbit IgG (Proteintech, Rosemont, IL, USA). Immune complexes were recovered using Protein A/G magnetic beads (MedChemExpress), washed, and reverse cross-linked at 65 °C for 4 h in elution buffer (0.1 M NaHCO_3_, pH 8.0, 1% SDS, and 200 mM NaCl) supplemented with proteinase K and RNase A. Isolated DNA was purified using DNA-binding magnetic beads (BioFACT, Daejeon, Republic of Korea) following the manufacturer’s protocol. Purified DNA was analyzed by quantitative real-time PCR on a CFX96 Real-Time PCR Detection System (Bio-Rad Laboratories) using primers specific for the proximal *CCL2* promoter region (−82 to +18 bp; Supplementary Table S1). ChIP-qPCR enrichment was first calculated as the percentage of input DNA (% input) for each immunoprecipitated sample. For graphical presentation, the % input values were subsequently expressed as fold change relative to the IgG control at 0 h, which was set to 1. IgG immunoprecipitation was performed at each time point as a negative control. Data were obtained from three independent chromatin preparations/ChIP experiments (n = 3).

### 4.8. House Dust Mite-Induced Atopic Dermatitis Murine Model

Male BALB/c mice (6–8 weeks old, 20–22 g; Orient Bio, Inc., Seongnam, Republic of Korea) were maintained under SPF conditions with a 12 h light/dark cycle, controlled temperature (22 ± 2 °C), and free access to standard chow and water. 

To induce AD-like skin inflammation, the ear skin of mice was treated with 4% SDS to disrupt the epidermal barrier, followed 2–3 h later by topical application of HDM extract cream (Biostir AD cream; 50 mg per ear). HDM was applied on days 1, 2, 3, 8, 10, 12, 14, 16, 18, and 20. For pharmacological treatment, IT25 or tofacitinib was formulated at 0.2% in 2.5% Pluronic F-127 and topically applied at 50 μL per ear on days 8–21. On days of HDM challenge, IT25 or tofacitinib was administered 30 min before HDM application. Control mice received the corresponding vehicle. Mice were sacrificed on day 22, and ear tissues were collected for subsequent histological and immunofluorescence analyses.

### 4.9. Histological, Immunohistochemical, and Immunofluorescence Analyses

Excised ear tissues were fixed in 4% paraformaldehyde, paraffin-embedded, and sectioned at 5 um.

Histopathology: Ear tissues collected on day 22 were sectioned and stained with hematoxylin and eosin (H&E). Total tissue thickness (epidermis plus dermis) was quantified from H&E-stained sections using ImageJ. Mast cells were visualized by staining with 0.1% toluidine blue (TB; Sigma-Aldrich).

Immunohistochemistry (IHC): Deparaffinized sections were subjected to heat-induced antigen retrieval in 10 mM citrate buffer (pH 6.0), quenched with 3% H_2_O_2_, blocked with 5% normal goat serum, and incubated overnight at 4 °C with primary antibodies against myeloperoxidase (MPO, 1:200; Abcam, Cambridge, UK) or F4/80 (1:200; Cell Signaling Technology). Sections were incubated with biotinylated secondary antibodies, developed with DAB substrate (Vector Laboratories, Burlingame, CA, USA), and counterstained with hematoxylin.

Immunofluorescence (IF): Deparaffinized sections were incubated with anti-CCL2 antibody (1:100; R&D Systems), and subjected to heat-induced antigen retrieval in basic buffer (10 mM Tris-HCl, pH 9.0, 0.05% Tween-20, and 1 mM EDTA) at 90 °C for 10 min. Endogenous peroxidase activity was blocked with 3% hydrogen peroxide for 10 min. The sections were then permeabilized with 0.3% Triton X-100 for 10 min at 25 °C and blocked for 1 h at 25 °C in TTBS containing 2.5% bovine serum albumin (BSA) and 2.5% goat serum, followed by Cy5-conjugated tyramide amplification (TSA Plus System, Akoya Biosciences, Marlborough, MA, USA) for 10 min. After washing with TBS, tissue autofluorescence was quenched by incubation for 10 min in a 1:1 mixture of ammonium chloride and copper sulfate. Nuclei were counterstained with 1 μg/mL Hoechst 33258 (Sigma-Aldrich). Images were acquired on a Nikon Eclipse Ti2-E fluorescence microscope (Nikon, Tokyo, Japan). Histological image acquisition and analysis were performed without blinding to treatment-group allocation.

### 4.10. Analysis of a Publicly Available Human Ad Transcriptomic Dataset

An RNA-seq gene-level raw count matrix comprising paired lesional and non-lesional skin biopsies from six patients with atopic dermatitis (AD) was obtained from the NCBI Gene Expression Omnibus (GEO; accession GSE140227). Lowly expressed genes were removed using filterByExpr, and raw read counts were normalized using the trimmed mean of M-values (TMM) method with edgeR (v4.10.1) in R (v4.6.0). Differential-expression analysis was performed using an edgeR quasi-likelihood generalized linear model with patient identity included in the design matrix (~Patient + Condition) to account for the matched lesional and non-lesional samples from each patient. *p* values were adjusted for multiple testing using the Benjamini–Hochberg method, and genes with an absolute log2 fold change (|log2FC|) ≥ 1 and a false discovery rate (FDR) ≤ 0.05 were considered differentially expressed. Volcano plots were generated using ggplot2 (v4.0.3). For visualization of individual genes, TMM-normalized log2 counts per million (log2CPM) were calculated using edgeR and plotted for paired lesional and non-lesional samples using GraphPad Prism (version 11.1.0). Differences in individual gene expression between paired samples were assessed using a two-tailed paired *t*-test. To ensure transparency and reproducibility, the analysis scripts, sample information, differential expression analysis results, and software environment details used for the reanalysis of GSE140227 are provided in the Supplementary Materials (Supplementary Files S1–S3, Supplementary Tables S3–S6).

### 4.11. Statistical Analysis

Data are presented as the mean ± standard deviation (SD) unless otherwise indicated. The number of independent biological replicates or experimental units is specified in the corresponding figure legends. Technical measurements, where applicable, were not treated as independent biological replicates. Statistical analyses were performed using GraphPad Prism (version 11.1.0). For comparisons involving more than two groups with a single experimental factor, one-way ANOVA followed by the multiple-comparisons test specified in the corresponding figure legend was used. For applicable one-way ANOVA analyses, homogeneity of variance was assessed using the Brown–Forsythe test; a non-significant result was interpreted only as no significant heterogeneity being detected and not as confirmation of equal variances, particularly given the limited sample sizes.

For experiments involving two experimental factors, two-way ANOVA was used, and interaction effects were evaluated where appropriate. For the *CCL2* promoter-mutagenesis experiment, promoter construct and TNFα treatment were analyzed as independent factors, followed by Tukey’s multiple-comparisons test. For the ChIP-qPCR time-course experiment, TNFα treatment time and ChIP antibody were analyzed as independent factors, followed by Dunnett’s multiple-comparisons test for α-EGR1 enrichment, with 0 h as the reference. Exact *p*-values are reported where informative, and *p* < 0.05 was considered statistically significant.

## 5. Conclusions

Our findings demonstrate that EGR1 contributes to TNFα-induced CCL2 expression in inflammatory keratinocytes. Using CCL2 as a representative candidate identified through exploratory transcriptomic profiling, we confirmed EGR1-dependent transcriptional regulation by complementary pharmacological, genetic, promoter–reporter, and ChIP analyses. The transcriptomic screen further identified expression patterns involving additional leukocyte-recruiting chemokines following treatment with EGR1 inhibitors; however, these observations remain exploratory and require independent biological validation. Together with the attenuation of HDM-induced skin inflammation by topical IT25, these findings support further investigation of EGR1 DNA-binding activity as a pharmacological target for modulating chemokine-associated inflammatory responses in the skin.

## Data Availability

The RNA sequencing data generated in this study have been deposited in the NCBI Gene Expression Omnibus (GEO) database under accession number GSE349203.

## Supplementary Materials

The following supporting information can be downloaded at: https://www.mdpi.com/article/10.3390/ijms27208973/s1.

## Abbreviations

AD	atopic dermatitis
CCL2	C-C motif chemokine ligand 2
ChIP	chromatin immunoprecipitation
EBS	EGR1-binding site
EGR1	early growth response 1
GO	Gene Ontology
GSEA	gene set enrichment analysis
HDM	house dust mite
IF	immunofluorescence
IHC	immunohistochemistry
JAK	Janus kinase
KEGG	Kyoto Encyclopedia of Genes and Genomes
MCP-1	monocyte chemoattractant protein-1
MPO	myeloperoxidase
MSigDB	Molecular Signatures Database
NF-κB	nuclear factor kappa B
PCA	principal component analysis
qPCR	quantitative polymerase chain reaction
RNA-seq	RNA sequencing
RT-PCR	reverse transcription polymerase chain reaction
shRNA	short hairpin RNA
TLR	Toll-like receptor
TNFα	tumor necrosis factor-α
