# Supplementary material for: Targeting EGR1 Attenuates CCL2 Expression in Inflammatory Keratinocytes and Allergic Skin Inflammation
Source: Int J Mol Sci. 2026 Oct 10;27(20):8973. doi: 10.3390/ijms27208973 (PMC13705188; doi:10.3390/ijms27208973)
Supplement: Supplementary file 1 [file ijms-27-08973-s001.zip › Supplementary Figure.pdf]

A

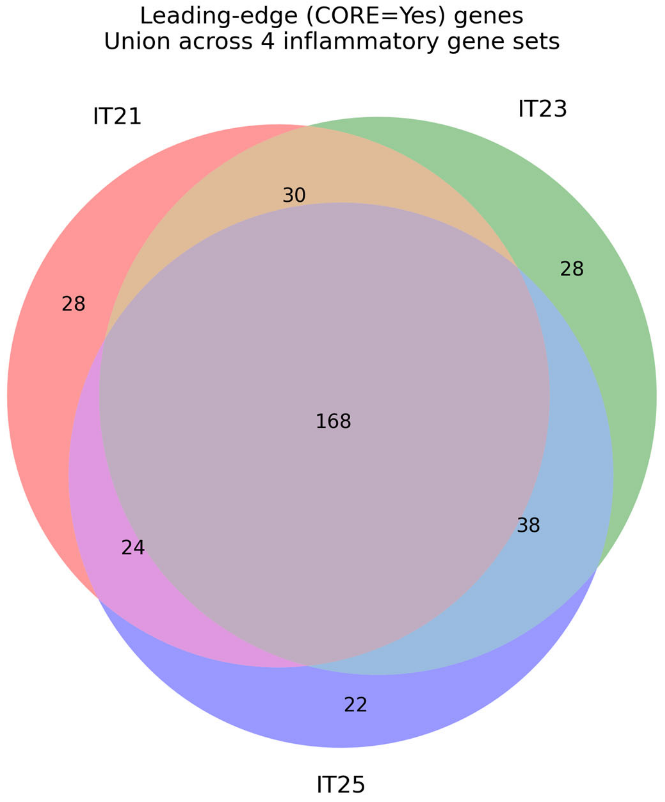

B

Gene	Geneset count (sum)	Geneset count (min)	Geneset count (max)	Category
CCL2	12	4	6	Chemokine
CCL5	9	3	5	Chemokine
IL6	8	3	4	Cytokine
RIPK2	7	2	5	Signal transduction / Innate immunity
CD40	7	2	4	Immune receptor
CDKN1A	6	2	4	Cell cycle regulator
CXCL11	6	2	4	Chemokine
CXCL9	6	2	4	Chemokine
GCH1	6	2	4	Metabolic enzyme
IL15	6	2	4	Cytokine
IL15RA	6	2	4	Cytokine receptor
IL18	6	2	4	Cytokine
INHBA	6	2	4	Cytokine
IRF7	6	2	4	Transcription factor
LIF	6	2	4	Cytokine
NFKBIA	6	2	4	Signal transduction / NF-κB regulator
TAP1	6	2	4	Antigen processing
TLR2	6	2	4	Pattern recognition receptor
NAMPT	6	2	4	Metabolic enzyme
ABI1	6	2	4	Signal transduction
BCL3	6	2	4	Transcription co-regulator
BTG2	6	2	4	Cell cycle regulator
CCL20	6	2	4	Chemokine
CCL22	6	2	4	Chemokine
EDN1	6	2	4	Secreted protein

Top 25 hub genes ranked by the cumulative frequency of occurrence across multiple pathway gene sets. Genes with geneset_count_sum ≥ 6 are shown.

Supplementary Figure S1. Prioritization of inflammatory hub genes by leading-edge gene integration.
(A) Venn diagram showing the overlap of leading-edge (CORE = Yes) genes extracted from four inflammatory Hallmark gene sets across IT21-, IT23-, and IT25-treated samples.
(B) Ranking of the top 25 candidate genes according to cumulative occurrence across enriched inflammatory gene sets (gene_set_count_sum). Genes were prioritized based on their repeated identification in multiple inflammatory pathways. CCL2 exhibited the highest cumulative frequency among all candidates. These analyses are descriptive and hypothesis-generating because RNA-seq was performed with one sample per experimental condition.

Kegg pathway

TNFα-upregulated genes vs untreated group

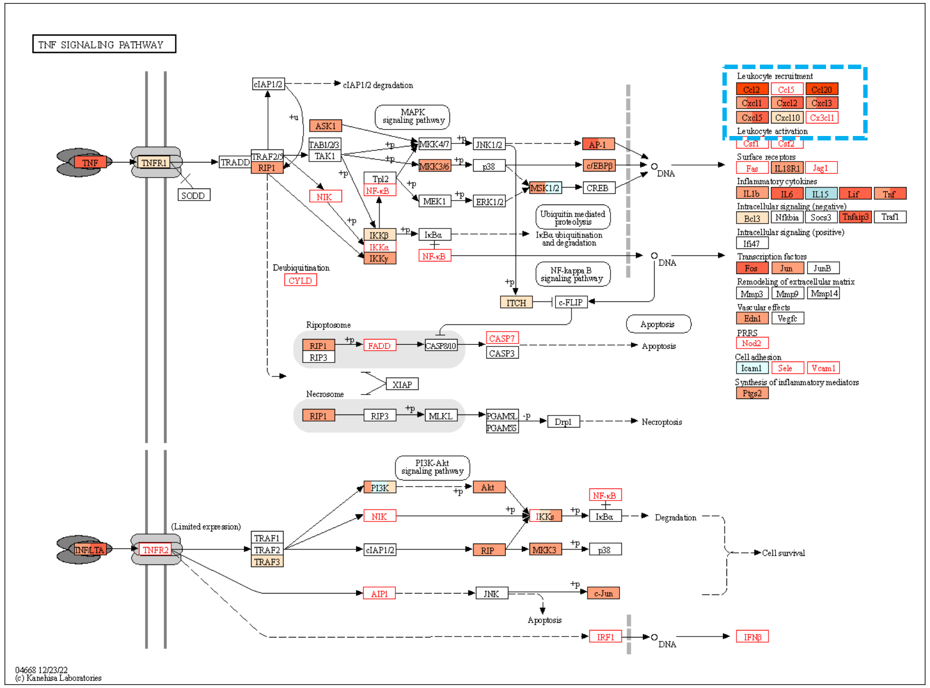

IT-mediated downregulation of TNFα-induced genes

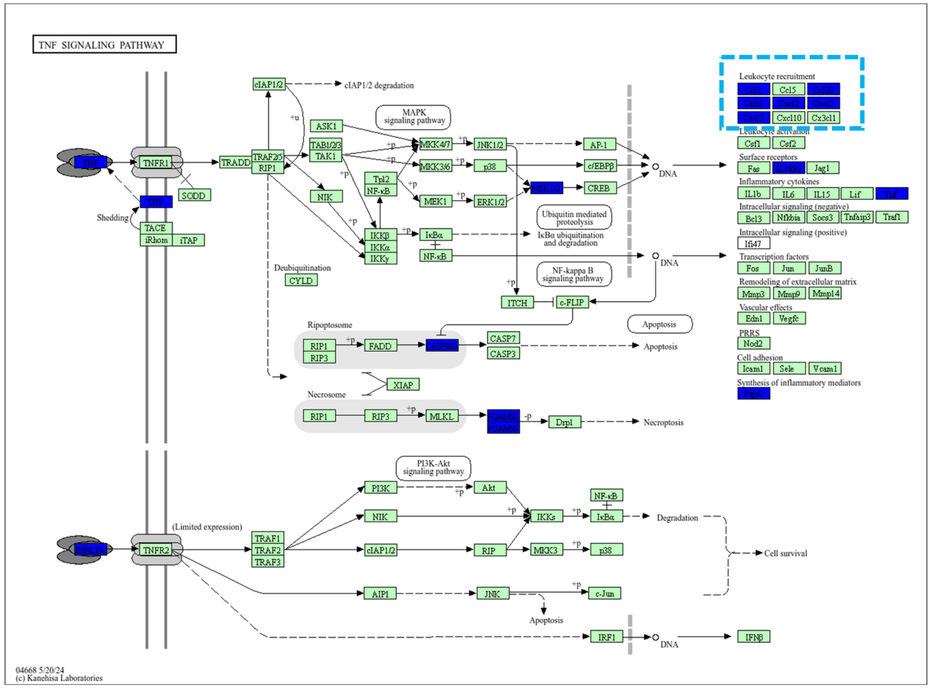

Supplementary Figure S2. KEGG pathway analysis identifies TNF signaling as a major inflammatory pathway suppressed by EGR1 inhibition. KEGG pathway enrichment analysis of TNFα-responsive and IT-responsive gene sets. Left, genes upregulated by TNFα relative to untreated controls. Right, TNFα-induced genes that were consistently downregulated following IT treatment. Genes are color-coded according to expression changes (red, upregulated; blue, downregulated), highlighting coordinated suppression of leukocyte recruitment-associated genes within the TNF signaling pathway. These analyses are descriptive and hypothesis-generating because RNA-seq was performed with one sample per experimental condition.

A

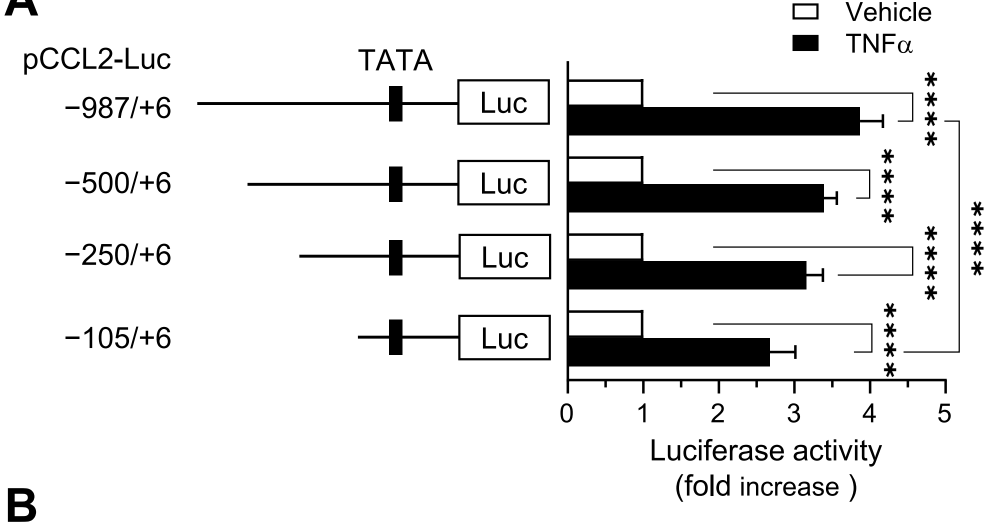

B

CCL2 (NM_002982.4)

```
−100   cccacttatc  actcatggaa  gatccctccct cctgcttgac  tCCGCCCTCT
                                                          EBS
− 50   Ctccctctgc  ccgctttcaa  taagaggcag  agacagcagc  cagaggaacc
                               TATA box
```

Supplementary Figure S3. Prioritization of inflammatory hub genes by leading-edge gene integration.
(A) Deletion mapping of the *CCL2* promoter. HaCaT cells were transfected with 5'-truncated luciferase reporter constructs (−987/+6 to −105/+6) and exposed to TNFα.
(B) Genomic sequence map of the minimal −105/+6 bp proximal promoter region, highlighting the putative consensus EGR1-binding site (EBS) and TATA box.
