# Supplementary material for: Targeting EGR1 Attenuates CCL2 Expression in Inflammatory Keratinocytes and Allergic Skin Inflammation
Source: Int J Mol Sci. 2026 Oct 10;27(20):8973. doi: 10.3390/ijms27208973 (PMC13705188; doi:10.3390/ijms27208973)
Supplement: Supplementary file 1 [file ijms-27-08973-s001.zip › Supplementary Table_S1-S2.pdf]

Targeting EGR1 Attenuates *CCL2* Expression in Inflammatory Keratinocytes and Allergic Skin Inflammation

Yena Choi[1,#], Euitaek Jung[1,#], Junekyu Han[1], Soon Young Shin[1,2,*]

[1]Department of Biological Sciences, College of Life Science, Konkuk University, Seoul 05029, Republic of Korea

[2]Cancer and Metabolism Institute, Konkuk University, Seoul, Republic of Korea

[#]These authors contributed equally to this work.

[*]Corresponding author. Soon Young Shin, E-mail, shinsy@konkuk.ac.kr (S.S. Shin)

Key words: Early growth response 1 (EGR1); CCL2; chemokine network; keratinocyte; skin inflammation; house dust mite; RNA sequencing; transcription factor inhibitor

Supplementary Materials

Contents

Supplementary Tables S1 page 2

Supplementary Tables S2 page 3

Supplementary Table S1. Primer sequences used for CCL2 promoter construct generation, RT-PCR, and qPCR**.**

**CCL2Ppromoter Construct**	**Primer sequence**	**PCR condition**
−987/+6	(F) 5'-GTCCACTGCTGCCTGCTATG-3'<br>(R) 5'-TTCCTCTGGCTGCTGTCTCT-3'	denaturation: 94°C, 30 s;<br>annealing: 60°C, 30 s;<br>elongation: 72°C, 1 min
−500/+6	(F) 5'-CTACAGTTCTGCTAGGCTTC-3'<br>(R) 5'-TTCCTCTGGCTGCTGTCTCT-3'	denaturation: 94°C, 30 s;<br>annealing: 60°C, 30 s;<br>elongation: 72°C, 30s
−250/+6	(F) 5'-ATCTGTGGTCAGTCTGGGCT-3'<br>(R) 5'-TTCCTCTGGCTGCTGTCTCT-3'	denaturation: 94°C, 30 s;<br>annealing: 60°C, 30 s;<br>elongation: 72°C, 30s
−105/+6	(F) 5'-GCAGATTTAACAGCCCACTT-3'<br>(R) 5'-TTCCTCTGGCTGCTGTCTCT-3'	denaturation: 94°C, 30 s;<br>annealing: 60°C, 30 s;<br>elongation: 72°C, 30s
**Site-directed mutagenesis**	**Primer sequence**	**PCR condition**
−105/+6 mtEBS	(F) 5'-ATCCCTCTGCCCGCTTTCA-3'<br>(R) 5'-ATAGGGCGGAGTCAAGCAGG-3'	denaturation: 94°C, 30 s;<br>annealing: 60°C, 1 min;<br>elongation: 72°C, 6 min
**ChIP-qPCR**	**Primer sequence**	**PCR condition**
EGR1 Target (-82/+18)	(F) 5’-AAGATCCCTCCTCC GCTTG-3’<br>(R) 5’-GGTTAGTCTCAGCCTCTCGG-3'	denaturation: 94°C, 20 s;<br>annealing: 60°C, 20 s;<br>elongation: 72°C, 30 s
**RT-PCR**	**Primer sequence**	**PCR condition**
**EGR1**	(F) 5'-CAGCAGTCCCATTTACTCAG-3'<br>(R) 5'-GACTGGTAGCTGGTATTG-3'	denaturation: 94°C, 30 s;<br>annealing: 58°C, 30 s;<br>elongation: 72°C, 30 s
**CCL2**	(F) 5'-CTTCTGTGCCTGCTCATAG-3'<br>(R) 5'-GTTTGGGTTTGCTTGTCCAG-3'	denaturation: 94°C, 30 s;<br>annealing: 62°C, 30 s;<br>elongation: 72°C, 30 s
**GAPDH**	(F) 5'-ACCCACTCCTCCACCTTT G-3'<br>(R) 5'-CTCTTGTGCTCTTGCTGGG-3'	denaturation: 94°C, 30 s;<br>annealing: 58°C, 30 s;<br>elongation: 72°C, 30 s
**qPCR**	**Primer sequence**	**PCR condition**
**EGR1**	(F) 5'-AGTTTGCCAGGAGCGATGAA-3'<br>(R) 5'-GGGACGGGTAGGAAGAGAGA-3'	Initial denaturation: 95°C, 3 min;<br>denaturation: 95°C, 10 s;<br>annealing: 55°C, 10 s;<br>extension: 72°C, 30 s
**CCL2**	(F) 5'-AGAATCACCAGCAGCAAGTGTCC-3'<br>(R) 5'-TCCTGAACCCACTTCTGCTTGG-3'	denaturation: 95°C, 3 min;<br>denaturation: 95°C, 10 s;<br>annealing: 55°C, 10 s;<br>extension: 72°C, 30 s
**GAPDH**	(F) 5'-GGAGCGAGATCCCTCCAAAAT-3'<br>(R) 5'-GGCTGTTGTCATACTTCTCATGG-3'	denaturation: 95°C, 3 min;<br>denaturation: 95°C, 10 s;<br>annealing: 55°C, 10 s;<br>Extension: 72°C, 30 s

Supplementary Table S2. List of the antibodies used in the study.

Antibody	Manufacturer	Catalog No.	Dilution
EGR1	Abcam<br>(Cambridge, UK)	ab194357	WB, 1:10000<br>ChIP, 1:100
EGR1	Proteintech<br>(Rosemont, IL, USA)	22008-1-AP	IHC, 1:500
GAPDH	Santa Cruz Biotechnology<br>(Dallas, TX, USA)	sc-32233	WB, 1:5000
CCL2	Abcam<br>(Cambridge, UK)	ab315478	IHC, 1:500
MPO-7	Cell Signaling Technology<br>(Danvers, MA)	#70076	IHC, 1:200
F4/80	Dako<br>(Carpinteria, CA)	A0398	IHC, 1:500

IHC, immunohistochemistry; WB, western blot.
